# Do single people age faster? The answer may lie in sleep

**DOI:** 10.3389/fragi.2025.1521401

**Published:** 2025-09-18

**Authors:** Jie-Yu Chuang

**Affiliations:** Mailman School of Public Health, Columbia University, New York, NY, United States

**Keywords:** aging, sleep, marriage, singlehood, single, longevity, anti-aging

Romantic relationship quality has been related to physical health, mental health, diet and physical activity (Sailer et al., 2024). Indeed, it would be optimal to have a supporting romantic relationship. However, global marriage rate is declining (https://ourworldindata.org/marriages-and-divorces). Furthermore, infidelity in romantic relationship is also increasing. Back in the year 2000, only about 8% of Chinese people were sexually unfaithful to their partners whereas by 2015, this rate had jumped to more than 24% (Luo and Yu, 2022). Evolutionarily speaking, how does this trend of subpar romantic relationship impact on human species? Moreover, will subpar romantic relationship affect human population health or even longevity?

Although not receiving much attention in previous researchers, the influence of romantic relationship on aging is an important consideration (Sailer et al., 2024). Despite the widely acclaimed benefits of being single, a significant body of research has supported the link between satisfactory romantic relationship and successful aging. In 1858, the famous epidemiologist William Farr suggested the longevity advantage of marriage in French men (Gellatly and Störmer, 2017). A recent longitudinal study (N = 7,641) indicates men who were continuously married aged more successfully (physical wellness, mental wellness, social wellness, and self-rated wellness) than their never-married counterparts (Ho et al., 2024). Furthermore, another longitudinal study (N = 974) with participants reported their romantic status at four phases (age 26, 32, 38 and 45) points to the association between slower biological aging (19 biomarkers and facial aging) and high-quality romantic relationship in both men and women (Bourassa et al., 2020). Marriage was also found to be associated with a slower epigenetic aging (Rentscher et al., 2023).

Nevertheless, the relationship between romantic relationship status and aging is complex. Singlehood has not been consistently associated with accelerated aging. Using data from the 2016 US Health and Retirement Study (aged 50–100, N = 3,765), Yu et al. failed to find prominent epigenetic aging difference between single and married people after controlling for sociodemographic characteristics, economic resources, depressive symptoms, and health behaviors (Yu, 2023). Furthermore, if marriage does indeed slower aging process, what is the underlying mechanism? A secure romantic relationship, rooted in attachment theory, may foster a sense of security, elevate Oxytocin levels, thereby mitigating the stress response and bolstering immune function (Maunder and Hunter, 2008). However, recent study failed to identify significant difference in mental and physical health between single and married population (Apostolou et al., 2024). Another study found that the odds ratio of meeting the WHO physical activity recommendations was 40% higher in single than in married participants (Puciato and Rozpara, 2021). Presumably, potential moderators or confounders in the relationship between marriage and aging should be considered.

Overall mental wellbeing has been shown to have a more prominent impact (1.65 years) on biological aging than the marital status (0.59 years) alone (Galkin et al., 2022). Indeed, researchers have found that with increasing age, having a partner is less predictive of psychological health and the satisfaction with being single increased (Böger and Huxhold, 2020). Among all psychological factors, subjective quality of sleep (0.44 years) has the largest impact on aging (Galkin et al., 2022). Moreover, a recent large cohort study (N = 589) suggests an association between poor sleep quality and accelerated brain aging, even in midlife (Cavaillès et al., 2024). In a sample of 154 middle-aged to older adults, it has been found that decelerated cellular aging (represented by telomere length) is associated with better sleep quality (assessed via Pittsburgh Sleep Quality Index) (Cribbet et al., 2014). Irrespective of marital status, sharing a bed with a partner has been linked with better sleep quality (Drews et al., 2020). Indeed, being married alone does not guarantee a good sleep quality since sleep quality was found to be correlated with couple relationship quality in a meta-analysis (Wang et al., 2025). No significant difference in sleep quality was found between married and single U.S. (Chen et al., 2015) and Japanese people (Matsumoto et al., 2022). Consequently, based on the scientific evidence, sleep quality may act as an unaddressed mediator in the intricate relationship between marriage status and aging.

As global trends indicate a staggering increase in both singlehood and the aging population, further comprehensive research is imperative and warranted to thoroughly investigate the complex interplay between aging and the dynamics of romantic relationships. Mental wellbeing, especially subjective sleep quality, was found to be significantly associated with aging. Based on current evidence, subjective sleep quality can be assumed to moderate the relationship between marital status and aging. Future research should explore the interplay between subjective and objective sleep quality, biological aging, and marital status, alongside other mental and physical health indicators, potentially through a prospective cohort study. If, as predicted, sleep quality is the most pivotal factor mediating the relationship between marital status and aging, individuals without partners can still decelerate aging process through improvement of sleep quality. In addition to general strategies to improve sleep hygiene (i.e., keep a consistent sleep schedule), specific interventions tailored to this singlehood-sleep quality-aging pathway could be explored and investigated to optimize sleep quality and mitigate aging in single individuals. Examples may include: sleeping with a soothing stuffed toy or sleeping with an artificial intelligence (A.I.) voice companion. Future studies are warranted to find the most effective strategy.

Oscar Wilde, the world-renowned Irish poet once said “To love oneself is the beginning of a lifelong romance.” Indeed, even amidst the trend of singlehood and population aging, we can still embrace the journey of self-love and find romance in the pursuit of health and youth. Furthermore, the secret to the fountain of youth might be sleep.
